# Study of microstructure formation in epoxy based systems using small angle neutron scattering

**DOI:** 10.1039/d5ra05789b

**Published:** 2025-12-17

**Authors:** Natasha Shirshova, Leide P. Cavalcanti, Sarah Youngs, Valeria Arrighi

**Affiliations:** a Department of Engineering, Durham University South Road Durham DH1 3LE UK natasha.shirshova@durham.ac.uk; b Science Technology Facilities Council, ISIS Neutron and Muon Source, Harwell Campus Oxfordshire OX11 0QX UK; c Institute of Chemical Sciences, School of Engineering & Physical Sciences, Heriot-Watt University Edinburgh EH14 4AS UK

## Abstract

Structural/multifunctional electrolytes (SE) are an essential part of novel types of energy storage devices, structural supercapacitors and structural batteries. They are able to perform two functions simultaneously, conduct ions and withstand mechanical load. The most promising SEs consist of two independent phases, *i.e.* have a bicontinuous structure. Here the formation of such structures is discussed using an epoxy cured in the presence of ionic liquid, as an example. Using small angle neutron scattering (SANS) structural changes were monitored as a function of curing time. It was necessary to use two models to fit the SANS data, at short and long curing times, indicating evolution of structural features at different length scales, with curing. Increasing temperature leads to significant increase in the reaction rate but the same trend is observed in the SANS patterns with curing time. Independently of the curing temperature, polymer clusters, in the range 25–75 nm, size is depending on the temperature, form during the early stages. As curing progresses, the number of clusters first increases and then a decrease is observed and accompanied by evolution of a more complex structure. Addition of a multifunctional block copolymer resulted in a significant change of the curing process at longer curing times.

## Abstract 2

Using SANS for *in situ* monitoring of the microstructure formation for the epoxy-based structural electrolyte allowed to see the effect composition and temperature have on it.

## Graphical abstract

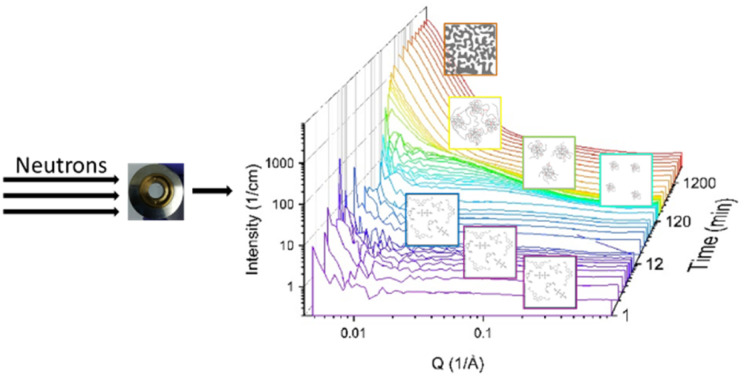

## Introduction

Multifunctionality is the ability of materials or devices to perform two or more functions simultaneously. One of the functions is usually structural and the other can vary widely from optical and antimicrobial to electrochemical.^1,2^ It is because of the variety of possible property combinations that multifunctional materials and devices continue to receive significant attention from the research community.^3–5^ Another important aspect is their ability to provide weight and/or volume savings, compared to materials with a single functionality.^6^ One of example of a multifunctional device is energy storage devices which combine structural function and energy storage ability.^5,7^ These devices offer significant potential in terms of weight savings in automotive and aerospace applications.

An energy storage device is considered truly multifunctional only when all its individual components, (electrodes, electrolyte and separator) are multifunctional, *i.e.* able to perform two functions simultaneously. Multifunctional electrolytes, also called structural electrolytes (SEs) are the most challenging component of such devices. Their complexity is in the inverse relationship that exists between two of main properties of SEs: ionic conductivity and mechanical performance.^8^

One of the most promising approaches for the preparation of SE reported in the literature is the formation of a bicontinuous system, where one phase is structural and responsible for the mechanical performance, while the other, containing a liquid electrolyte, determines the ionic conductivity.^5,9–11^ Due to a wide range of desirable properties, such as mechanical, thermal and chemical stability, epoxies are one of the best candidates for the structural part of the multifunctional electrolyte.^5,12^

Typically, SEs based on epoxies are synthesised using reaction or polymerisation induced phase separation (RIPS and PIPS, respectively).^5,13,14^ In both cases, an initial, one-phase multi-component mixture phase separates during the reaction. Chain growth results in an increase of the cross-link density, reduction of the solubility and, finally, formation of a two-phase structure. The composition of the initial reaction mixture affects the reaction mechanism as well as the morphology and properties of the resulting SE. So far, research has mainly focused on improving the SE's mechanical performance and ionic conductivity with very little attention paid to more fundamental aspects such as controlling properties through microstructure formation.

The link between microstructure and physical properties of thermosetting resins is well documented in the literature with size, density and interconnectivity of phase-separated domains playing an important role.^15–17^ However, successful attempts to control microstructure are still limited due to the complexity of the reaction mechanism which is affected by the composition of the initial reaction mixture, reaction temperature, as well as curing rate and post-curing.^18–21^ The type of microstructure depends on the rates of competitive reactions, *i.e.* phase separation and structural freezing by gelation caused by the polymerisation. It has been suggested^18,19^ that, if gelation occurs significantly earlier than phase separation, no macropores would be formed. If gelation occurs after phase separation, the bicontinuous phase breaks up forming a nodular structure to reduce interfacial energy.^19^ However, when both reactions run in parallel, a bicontinuous structure is obtained.

To be able to develop SEs with optimal properties it is essential that a relationship between network structure and physical properties can be established. Characterisation of the conventional network structure of thermosets is carried out using techniques such as scanning (SEM) and transmission electron microscopy (TEM), atomic force microscopy (AFM),^22,23^ small-(SAXS) and wide-angle X-ray scattering (WAXS), and small angle neutron scattering (SANS).^24–28^ However, as often discussed in the literature, it is usually not possible to undertake a direct investigation of how the network structure develops due to insolubility of thermosets in organic solvents.^29^ Indirect studies have been performed on fracture surfaces of cured epoxies using AFM and SEM.^24,30–32^ An indirect procedure to carry out SAXS and SANS measurements has also been developed. This involves curing samples for different lengths of time and performing scattering measurements after swelling to enhance the local fluctuations of the cross-link density and spatial inhomogeneity.^27,33,34^ As discussed later in the manuscript, the presence of the IL in our epoxy formulations, which can be deuterated, allows for direct, *in situ* SANS measurements giving an insight into the structural evolution of the network.

It was shown that SANS can be used to study bisphenol A diglycidyl ether (DGEBA) based formulations forming interpenetrating networks (IPNs).^35^ In this work, a deuterated dimethacrylate (bis-GMA) was used to increase the contrast between the two networks, formed by bis-GMA and DGEBA.^35^ Only fully cured formulations were studied using SANS. All formulations showed a two-phase structure with scattering intensity being dependent on composition, especially the amount of initiator used. Changes in the reaction rate led to changes in the gelation time of the bis-GMA and, consequently, the extent of phase separation and microstructure. A further study was reported in which curing of deuterated DGBEA with a series of amines was investigated. Once again, SANS experiments were carried out on fully cured samples.^36^

The SANS study reported here focuses on SEs based on DGEBA (here referred to as Ep) and the ionic liquid 1-ethyl-3-methylimidazolium bis (trifluoromethyl sulfonyl) imide (EMIM-TFSI, here referred to as IL) cured using isophorone diamine (iPDA). We show that the presence of an ionic liquid in SEs can be advantageously exploited to monitor the kinetics of curing, *in situ*, using SANS.

Qualitative SANS measurements reported by us in a recent publication^13^ demonstrated that addition of a block copolymer affects the nanoscale structure of structural electrolytes. Here the structure development during curing of Ep in the presence of the IL and models that describe the scattering data as a function of time, are discussed. The effect of curing temperature and composition of the initial reaction mixture on the structure formation is reported and analysed. A full characterisation of the block-copolymers in the IL solution is also reported.

## Experimental

### Materials

Bisphenol A diglycidyl ether (Ep), and hardener isophorone diamine (iPDA), were purchased from Sigma Aldrich. The ionic liquid, 1-ethyl-3-methylimidazolium bis(fluorosulfonyl)imide (EMIM-TFSI, here referred to as hydrogenated IL (h-IL) >99%), was purchased from Ionic Liquid Technologies (IOLITEC, Germany). All chemicals were used as received.

Deuterated IL (d-IL) was synthesised at the ISIS Deuteration Facility (Science Technology Facilities Council; ISIS Neutron and Muon Source; Harwell Campus; UK) using published procedure.^37^ The mass spectrometry analysis indicated a detailed isotopic distribution with a weighted average of approximately 10.03 deuteriums and an overall deuteration level of ∼91.2%. The calculation was performed using the software DGET!^38^ The full mass profile between *m*/*z* 200–300 with peak labels provides a complete visualization of the sample and are presented in the SI (Fig. S1). ^1^HNMR of the d-IL was run in *d*-DMSO and can be found in Fig. S2.

**Scheme 1:**
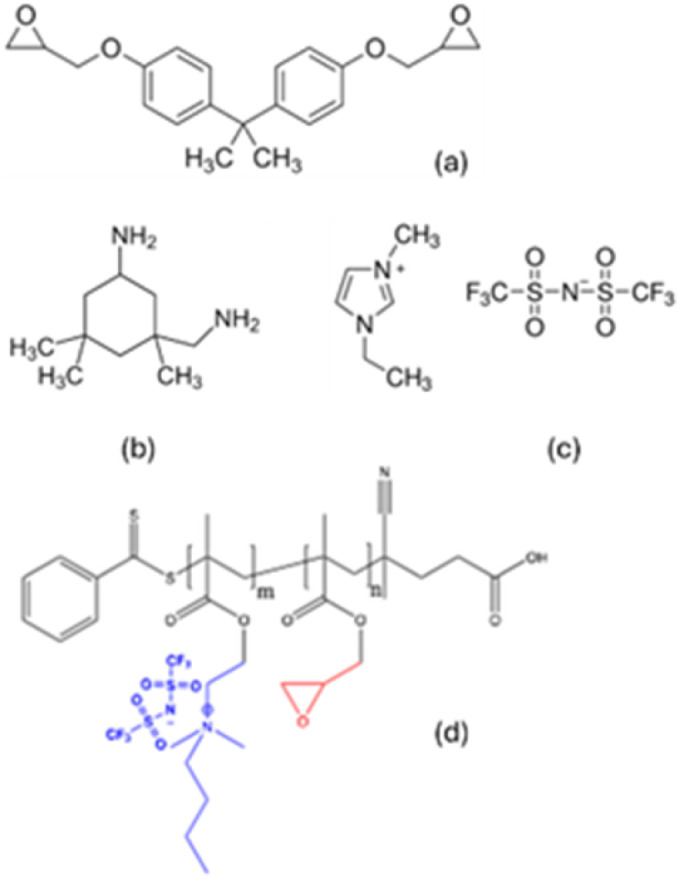
Chemical structures of compounds used in this study: (a) DGEBA (Ep); (b) iPDA; (c) h-IL; (d) general structure of the multifunctional block-copolymer (pGMA)_*n*_-*block*-*co*-p(DMAEMA-TFSI)_*m*_, where *n* = 35 and *m* = 7.

The block-copolymer, poly(glycidyl methacrylate)-*block*-poly[(2-dimethylamino)ethyl methacrylate) butyl bis(trifluoromethane)sulfonimide] (pGMA)35-*b*-p(DMAEMA-TFSI)17, herein after referred to as MF-bcP, was synthesised according to a procedure reported elsewhere.^13,39^ The structure of the multifunctional block-copolymer as well as all structures of other chemicals used in this study are shown in Scheme 1.

### Preparation of the structural electrolyte

For samples without MF-BcP, Ep was mixed with d-IL or h-IL, followed by iPDA. The mixture was then stirred until a homogeneous solution was formed, at which stage it was degassed using a sonication bath. For the samples with MF-bcP, 1wt% of MF-bcP was added to the EP (amount of MF-bcP was calculated as wt% to the amount of Ep + IL), followed by addition of EMIM-TFSI. The resulting mixture was then stirred until the polymer was fully dissolved using a roller mixer (SciLogex MX-T6-S) at 30 rpm. iPDA was then added to the solution and the mixture was stirred and degassed. The compositions of the studied formulations are summarised in Table 1.

**Table 1:** Compositions of formulations used for the SANS experiments

Sample	DGEBA, g	IPDA, g	d-IL, g	MF-bcP, g	Curing temperature, °C
Ep50dIL^a^	0.1	0.025	0.058	0	r.t.
Ep_r.t.	0.1	0.025	0.115	0	r.t.
Ep_50°	0.1	0.025	0.115	0	50
Ep_60°	0.1	0.025	0.115	0	60
Ep/MF-BcP	0.1	0.025	0.115	0.006	r.t.

a Mixture of 0.058 g of h-IL and 0.058 g of d-IL were used to prepare this formulation.

### Small angle neutron scattering measurements

The SANS measurements were carried out on SANS2D and ZOOM at the STFC ISIS Pulsed Neutron source (Rutherford Appleton Laboratory, Didcot UK). The *Q* range (*Q* = (4π/*λ*) sin(*θ*/2), where *θ* is the scattering angle and *λ* the neutron wavelength), explored on ZOOM varied from 0.0025 to 0.5 Å^−1^ whereas on SANS2D the *Q*-range was 0.0015 to 0.5 Å^−1^.

Three types of measurements were carried out: (a) on a series of background samples, (b) on a solution of MF-bcP, to study its conformation and possible self-assembly behaviour and (c) on the formulations presented in Table 1, to monitor the RIPS curing kinetics. Hellma liquid cells were used for all background samples and solutions of Mf-bcP in the d-IL. A modified Durham (gel) cell was used to study the curing kinetics. Samples were sandwiched between two quartz windows and an aluminium spacer was used to give a sample thickness of 1 mm and a diameter of 10 mm.

Background samples included (1) Ep:d-IL 60 : 40 vol%; (2) iPDA:d-IL (as for formulation with 40 vol% IL); (3) hydrogenated IL (h-IL); (4) d-IL; (5) an empty cell. These samples showed no visible structural features and only background scattering was measured.

To characterise the MF-bcP, SANS measurements were carried out in a solution containing h-IL and d-IL. As shown in Fig. S3 of the SI, a 20 mg ml^−1^ solution of MF-bcP in the h-IL gave no structural information, due to the lack of contrast between the block-copolymer and the h-IL. All subsequent measurements were therefore performed using the d-IL.

All scattering data were normalised for sample transmission and the incident wavelength distribution, corrected for instrumental and sample backgrounds using an empty cell. The scattered intensity was converted to the normalized differential scattering cross section per unit volume, dΣ(*Q*)/dΩ, expressed in units of cm^−1^ using a blend of hydrogenated and deuterated polystyrene standards.

Neutrons are scattered by short-range interactions with nuclei within the sample. The ‘scattering power’ is defined by the scattering-length density (SLD), *ρ* (cm^−2^), which can be calculated from:1
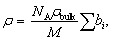
where *N*_A_ is Avogadro's number, *M* is the molecular weight of the scattering centre (a particle or molecule) and *ρ*_bulk_ is the corresponding density. As indicated in eqn (1), the average atomic scattering lengths bi are summed over the whole particle/volume; SLD values for chemicals used in this study can be found in Table S1.

The differential scattering cross section dΣ(*Q*)/dΩ is a function of the scattering vector or momentum transfer *Q*. dΣ(*Q*)/dΩ (indicated as *I*(*Q*) later) can be written in terms of the form factor *P*(*Q*) and the structure factor *S*(*Q*), as:2
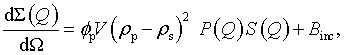
where *ϕ*_p_ is the volume fraction of polymer, *V* is the volume of a polymer coil. The terms *ρ*_p_ and *ρ*_s_ represent the scattering length densities of the polymer, and the solvent (which in the case of this study is IL), respectively. Thus (*ρ*_p_ − *ρ*_s_)^2^ is the contrast. The incoherent scattering, *B*_inc_, can either be subtracted from the data using appropriate background measurements or added to the model as an adjustable fitting (constant) parameter. The form factor, *P*(*Q*), is a dimensionless function that accounts for interference effects between neutrons scattered from different parts of the same scattering entity. It defines the size and shape of the scattering object. The *S*(*Q*) is the interparticle structure factor which describes positional correlations between particles/objects.

## Results and discussion

### Time dependence of structural changes during RIPS

The three-dimensional network, formed during the reaction between oxirane groups of an epoxy resin and the hydrogen of the amine, is reported to display structural inhomogeneities.^40,41^ Those inhomogeneities are commonly attributed to two main factors: (a) cross-links which fix the topology of the network structure and (b) the kinetics of crosslinking which introduces a non-random distribution of crosslink-rich and -poor regions as well as network defects such as loops and dangling chains.

On the basis of the previous study,^13^ here the curing kinetics of an epoxy-based formulation containing 40 vol% IL were monitored. SANS measurements were carried out at room temperature (r.t.) in a mixture of the h-IL and d-IL to enhance contrast, at first to reduce use of the deuterated material, and later using pure d-IL for consistency. After addition of iPDA, the SANS intensities were recorded over a period of *ca.* 12 hours and, in order to carry out a kinetic analysis, data were subsequently sliced at required intervals. The experiment was carried out using ZOOM with the minimum *Q* value of 0.0025 and the *d*-spacing (≈2π/*Q*) from 2512 Å^−1^.

**Fig. 1:**
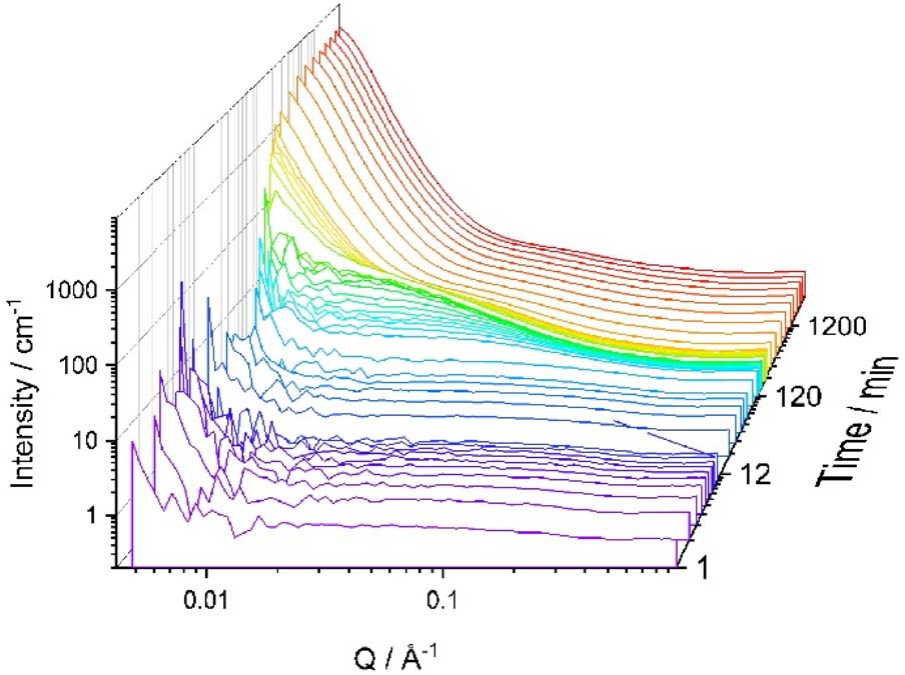
SANS data for EP50dIL as a function of curing time. The sample consists of epoxy and iPDA in a mixture of d- IL:h-IL (50 : 50 wt%). EP:iPDA = 4 : 1 wt./wt.; [IL] = 40 vol%.

Fig. 1 shows the evolution of the SANS patterns of the EP50dIL sample. The formulation is initially homogeneous, but phase separation occurs during curing as the growing network becomes insoluble in the liquid electrolyte, *i.e.* the ionic liquid.

The SANS traces up to 160 min are characterised by an upturn at *Q* values below 0.01 Å^−1^, followed by a broad shoulder in the intermediate *Q* range from 0.01 to 0.5 Å^−1^ and a plateau above 0.5 Å^−1^. The behaviour is qualitatively similar to that observed in other cross-linked systems such as swollen polymer gels, thermosets^26,27,40,42,43^ and cellulose nano-sponges.

To extract quantitative information from the SANS data, models developed for cross-linked polymer gels and later applied to thermosets^26,27,40,42^ were considered. Phenomenologically, the scattering of these systems can be described as a combination of long-range frozen inhomogeneities and local liquid-like fluctuations. Thus, the total scattering, *I*(*Q*)_tot_, is made up of two separate contributions, plus a *Q* independent background, *B*_inc_, largely due to incoherent scattering:

3 *I*(*Q*)_tot_ = *I*(*Q*)_L_ + *I*(*Q*)_s_,

were *I*(*Q*)_L_ and *I*(*Q*)_s_ refer to the large- and small-scale structures observed at low and high *Q*, respectively. In the literature, the former have been assigned to long-range static inhomogeneities whereas the latter have been associated with local liquid-like or thermal fluctuations.^41,44–47^

**Fig. 2:**
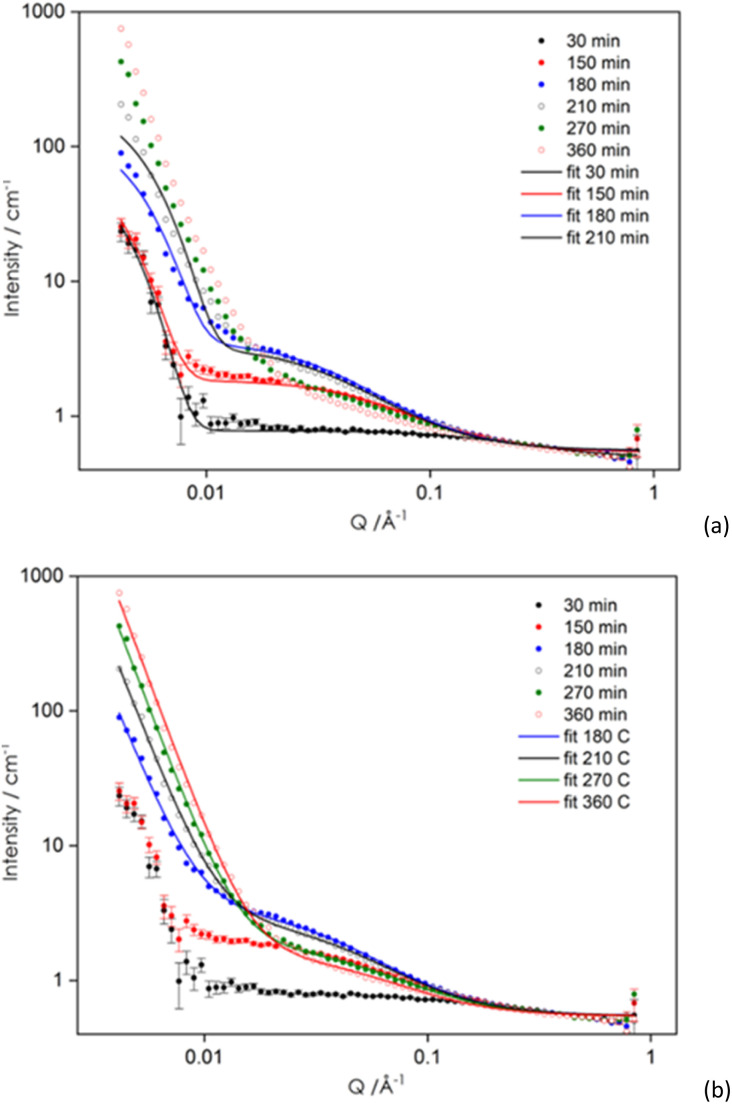
SANS data of EP50dIL during curing. Symbols represent experimental data. (a) Lines are fits to the experimental data using eqn (4). (b) Lines correspond to fits using the correlation length model, eqn (7). Curing was done in a mixture of d- IL:h-IL (50 : 50 wt%).

For *Q* < 0.010 Å^−1^, in the first hour of curing, the *I*(*Q*) data seem to display a peak whose maximum is of the order of the lowest experimentally accessible *Q* value (Fig. 2). The existence of distinct scattering peaks has been reported for polymer gels and attributed to microphase separation due to large concentration fluctuations with polymer-rich and -poor domains. From the SANS data presented in Fig. 2, the repeat distance of the concentration fluctuations, that is, 2/*Q*_*m*_, where *Q*_*m*_ is the magnitude of the scattering vector at the scattering maximum, is of the order of 40 nm.

Practically no change in the size of the static inhomogeneities at *Q* < 0.010 Å^−1^ is observed during the first few hours of curing, with only small variations in the extent of liquid-like fluctuations. Upon further curing, the shoulder at high *Q* moves towards lower *Q* values, indicating an increase in the size of the characteristic domains, as well as their number (Fig. 2). At *Q* < 0.010 Å^−1^, the scattered intensity approximately follows Porod's law (*Q*^4^ dependence) as expected for two-phase systems with large domains.^33^ The shift towards higher *Q* with curing suggests a decrease in the size of the phase separated structure.

The scattering from a gel structure, but typically a physical rather than chemical network, can be modelled as the sum of a low-*Q* exponential decay (which happens to give a functional form similar to Guinier scattering) plus a Lorentzian at higher-*Q* values.

In the early stages of the curing process, the following relationship can be used to fit the SANS data4
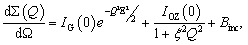
where the first term corresponds to an expression for *I*(*Q*)_L_ in eqn (3) and results from the built-in inhomogeneity due to formation of cross-links. Therefore, Ξ represents the length scale of the solid-like (static) correlations. The second term, which corresponds to *I*(*Q*)_s_ of eqn (3), is the Ornsteine–Zernike (OZ) equation, often used to describe the scattering of polymer solutions. *I*_G_(0) and *I*_OZ_(0) quantify the relative contributions, *i.e.* the scattering intensity at *Q* = 0, of the two terms in eqn (4). *B*_inc_ is a *Q* independent background, largely due to incoherent scattering and *ξ* is a shorter correlation length.

**Fig. 3:**
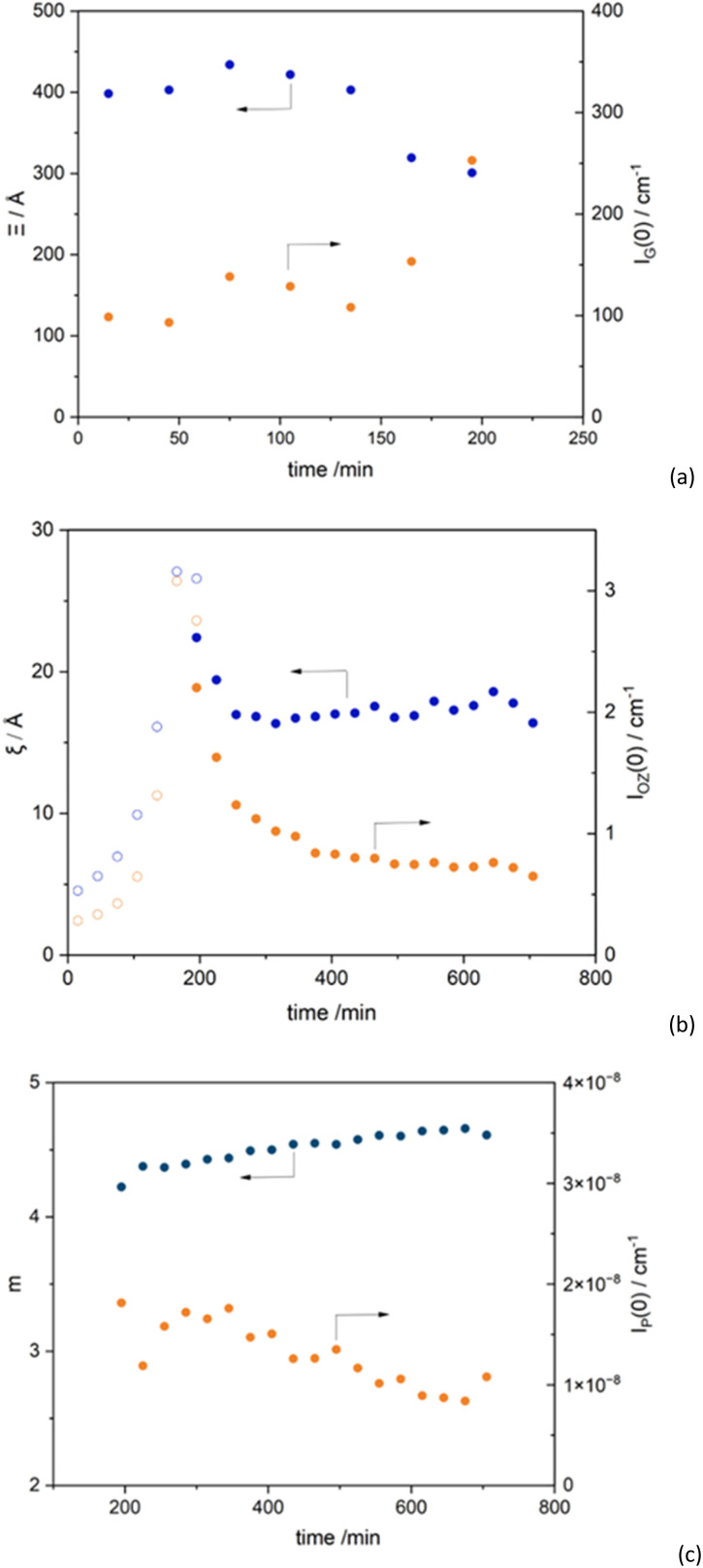
Fitting parameters obtained from the SANS data of EP50dIL as a function of curing time. (a) Large scale correlation length, Ξ, and scale parameter, *I*_G_(0), from eqn (4); (b) correlation length, *ξ* and scale parameter *I*_OZ_(0) of the Lorentzian component as obtained from fits using eqn (4) (empty symbols) and eqn (7) (filled symbols); (c) Porod exponent (*m*) obtained from the fits using eqn (7) and *I*_P_(0), representing A in the eqn (7).

It was found that the eqn (4) adequately described the data in the early stages of the reaction, up to 180–210 min, as shown in Fig. 2(a). It can be observed (Fig. 3(a)) that the large-scale correlation length Ξ is nearly constant with values being in the range 282 to 307 Å, whereas *ξ* values vary from 4.5 to 27 Å, following a *t*^3.4^ dependence (Fig. 3(b)). As curing time increases above 210 min deviations at low *Q* become more pronounced (Fig. 2). This means that to extract quantitative information from the SANS data, different models need to be used, at short and long curing times.

According to previous SAXS and SANS studies, inhomogeneities and concentration fluctuations in polymer gels are well represented by the squared-Lorentzian^48^ (*I*_SL_(*Q*)) and the Ornsteine–Zernike equations, respectively, leading to:5
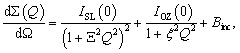
where *I*_SL_(0) is the squared-Lorentzian scale parameter.

The statistical theory of Panyukov and Rabin^49^ which accounts for both thermal and frozen concentration fluctuations in polymer gels, identifies the first and second terms in eqn (5) as the frozen and thermal structure factors, respectively.

Improved fits can often be achieved by accounting for a more pronounced decay of the scattered intensity at low *Q* and therefore allowing the exponent, *n*, of the squared Lorentzian (eqn (5)) to take values higher than 2:6
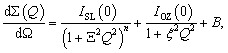
where *I*_SL_(0) is the squared-Lorentzian scale parameter.

While fitting data using eqn (5) or (6) generally provided consistent values of correlation lengths, ξ, from the Ornsteine–Zernike component, the Ξ values and scale factors were found to be highly correlated (as evidenced by very large errors in these two fitting parameters). This is likely due to the fact that the size of the structures formed in the epoxy network during curing are larger than the length scale probed, as well as uncertainties in the small *Q* region.^50^ As the result, the first terms in eqn (5) and (6) have reached their asymptotic limit.

A model proposed by Hammouda *et al.* was used to fit the data consisting of a Debye–Bueche-type squared-Lorentzian function^51^ and a power-law function:^52^7
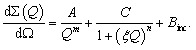


The first term in the above equation describes Porod scattering, with *m* being a Porod exponent, and the second term is a Lorentzian function describing, in this case, scattering from thermal fluctuations.

Fitting the SANS data using eqn (7) was successful for the curves recorded for curing times longer than 180 min (Fig. 2(b)). Despite having used two different models to fit the data at short and long curing times, the *ξ* values in (Fig. 3(b)) are continuous, showing an increase at short curing time, followed by a decrease. A similar pattern for the *ξ* values with time was observed by Izumi *et al.* in their SAXS/WAXS study of phenolic resins.^40^ These, authors related changes in the values of *ξ* to the average size of soluble oligomers and mesh size of the gel network, which is believed to behave as a polymer chain in the semidilute regime. Thus, the observed increase/decrease in the *ξ* value with reaction time was attributed to an increase in the size of soluble polymer chains and a decrease in the mesh size, respectively. The observed behaviour can be further explained by the formation of the polymer clusters at the early stages of the reaction and a sharp rise in their numbers, followed by the decrease in numbers, as shown by the reduction in the *ξ* value and formation of the more complex structures.

Changes in the correlation length, Ξ, values with time in the early stages (up to 125 min) was less dramatic (Fig. 3(a)), in comparison to the shorter correlation length, *ξ*, related to the initial formation of the loosely crosslinked network. As cross-linking density of the system increases, the values of Ξ expectedly decrease. The observed trend correlates well with findings for other thermoset system, specifically, phenolic resins.^40^

**Fig. 4:**
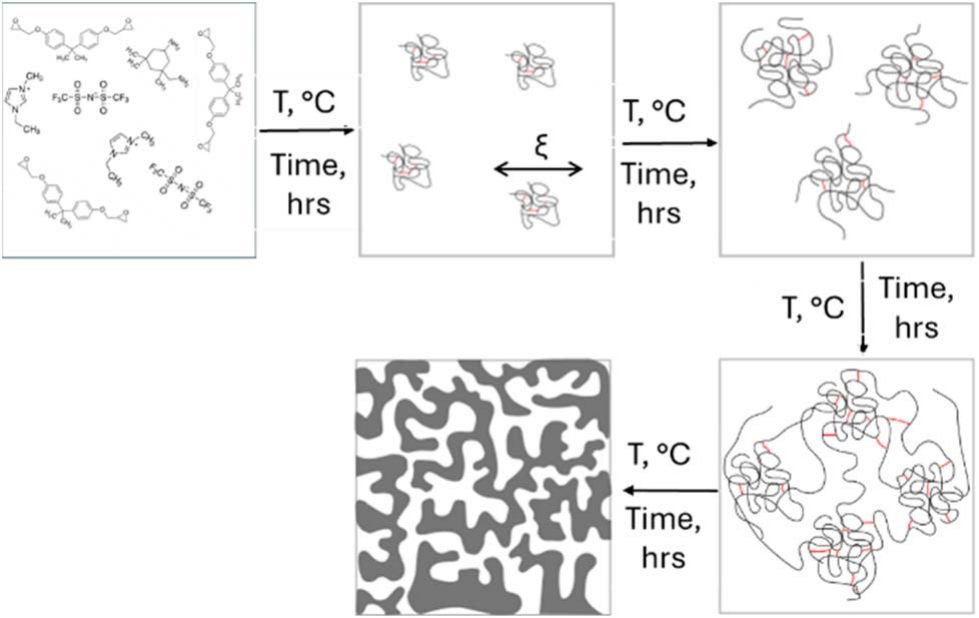
Schematic of the microstructure formation in epoxy-based formulations.

The trends observed between the two correlation lengths (Ξ and *ξ*) as a function of curing times, appear to support the idea that cross-linking starts within small clusters. Once a given size and number of clusters is reached, a percolating structure forms by merging of clusters (Fig. 4). These findings provide experimental support to the coarse grain molecular dynamics simulations of Kroll *et al.*,^53,54^ showing that cross-linking leads to formation of heterogeneous clusters that grow by incorporation of precursor molecules until, close to the gel-point, they coalesce.

Fig. 3(c) shows changes in the *m* values with curing time. A value of *m* = 4, indicates the existence of a two-phase structure with a sharp boundary (*I*(*Q*) ≈ *Q*^−4^). However, *m* values of the exponent greater than 4 are not uncommon and have been attributed to “fuzzy” surfaces.^55^

### Effect of the temperature on the structure formation

**Fig. 5:**
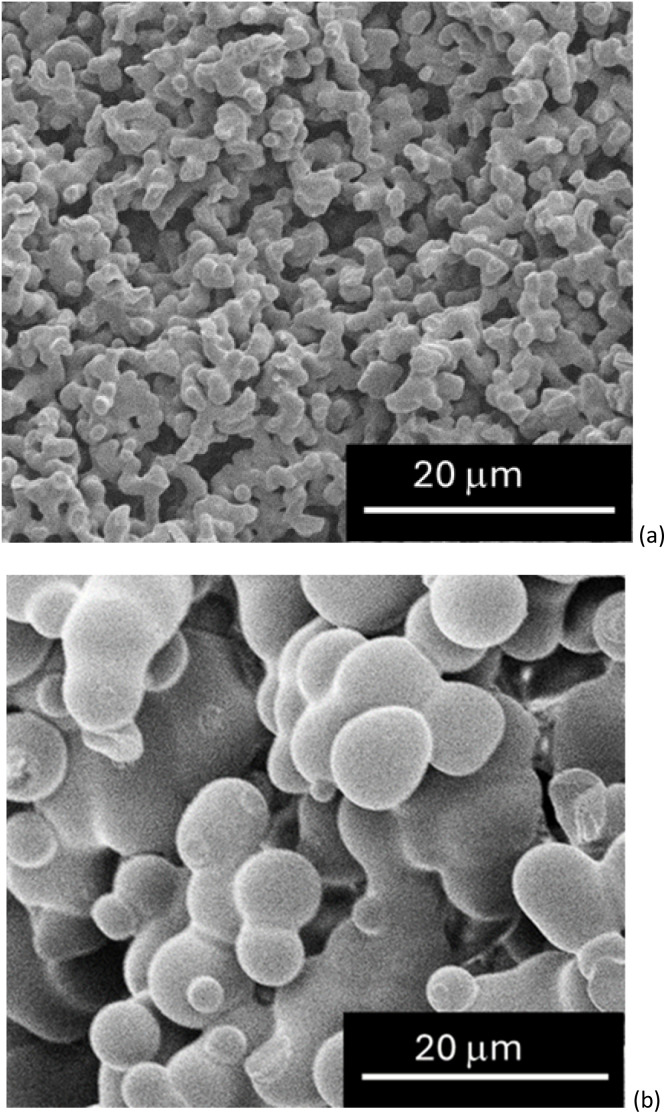
SEM micrographs of structural electrolytes cured using (a) r.t. cure and (b) 60 °C.

Previous research^13^ showed how temperature affects the morphology and properties of the DGEBA cured using iPDA in the presence of the h-IL. More specifically, an increase of the feature sizes and significant change in their shape was observed upon removal of the room temperature step from the curing cycle (Fig. 5). As shown in Fig. 5, samples cured at 60 °C developed a nodular structure nearly an order of magnitude larger (Fig. 5(b)) than the microstructure formed at room temperature (Fig. 5(a)). The homogeneity of the microstructure formed was also affected (Fig. 5) and samples cured including the room temperature step showed more homogeneous features. This is in agreement with data in the literature where, epoxy cured using amine at lower temperature, was shown to have less inhomogeneities in their structures.^56^

**Fig. 6:**
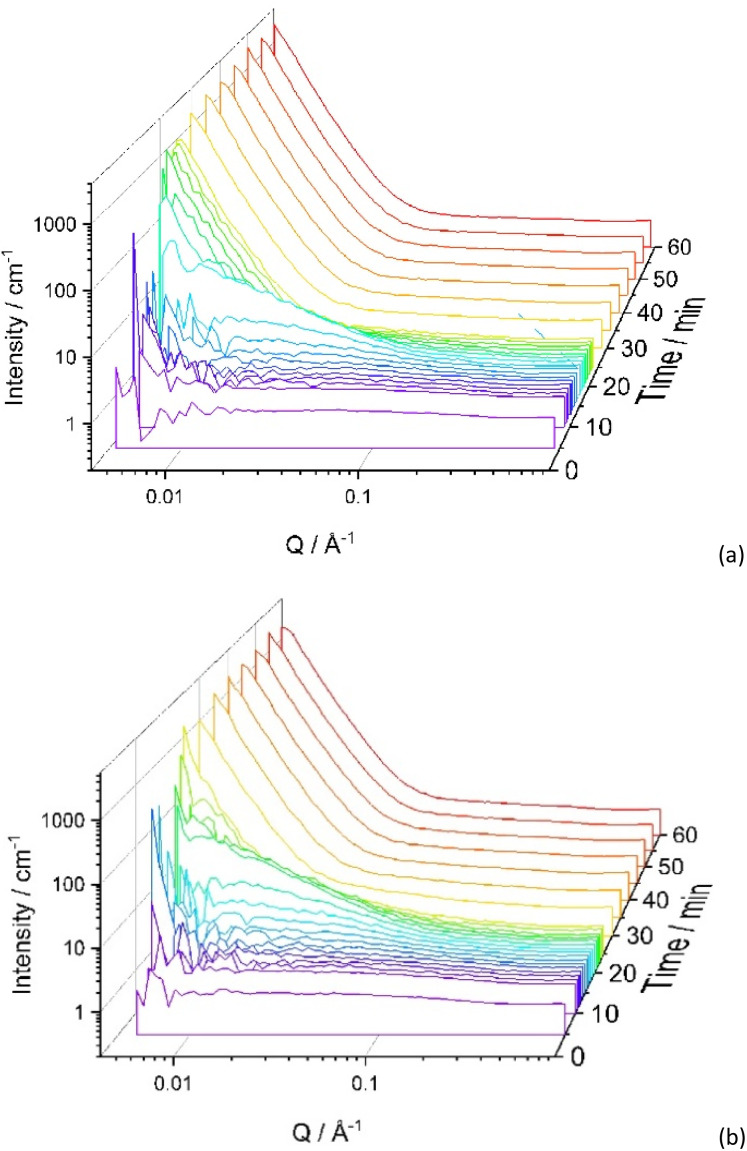
Effect of temperature on SANS data for Ep system. (a) 60 °C and (b) 50 °C. The sample consists of epoxy, iPDA in d-IL; EP:iPDA = 4 : 1 wt./wt.; [IL] = 40 vol%.

It was expected that SANS could help to assess the difference in structure formation and that is why SANS experiments were run at 60 °C using d-IL only (Sample Ep_60°) (Fig. 6(a)). The rate of the curing reaction was significantly faster than at r.t. and it was difficult to analyse the early stages of the reaction. As shown in Fig. 6(a), even after 5 min, the SANS curve has a very distinct upwards trajectory at low *Q*, indicating large scale inhomogeneity. However, the overall trend is similar to that observed for r.t. cure, *i.e.* in the earlier stages of the reaction the scattering at high *Q* (>0.25 Å^−1^) and mid *Q* values (0.1 < *Q* > 0.25) is nearly *Q* independent with an upturn is observed at the low *Q* values (<0.1). With increasing reaction time, at high and mid-range values of the *Q*, a shallow peak appears and the upturn in the low *Q* range was increased in intensity. To reduce the rate of the reaction, the temperature was lowered to 50 °C (Sample Ep_50°) (Fig. 6(b)). Once again, a trend similar to one presented in Fig. 6(a) was observed, while indicating that the system at 50 °C remains homogeneous for longer.

**Fig. 7:**
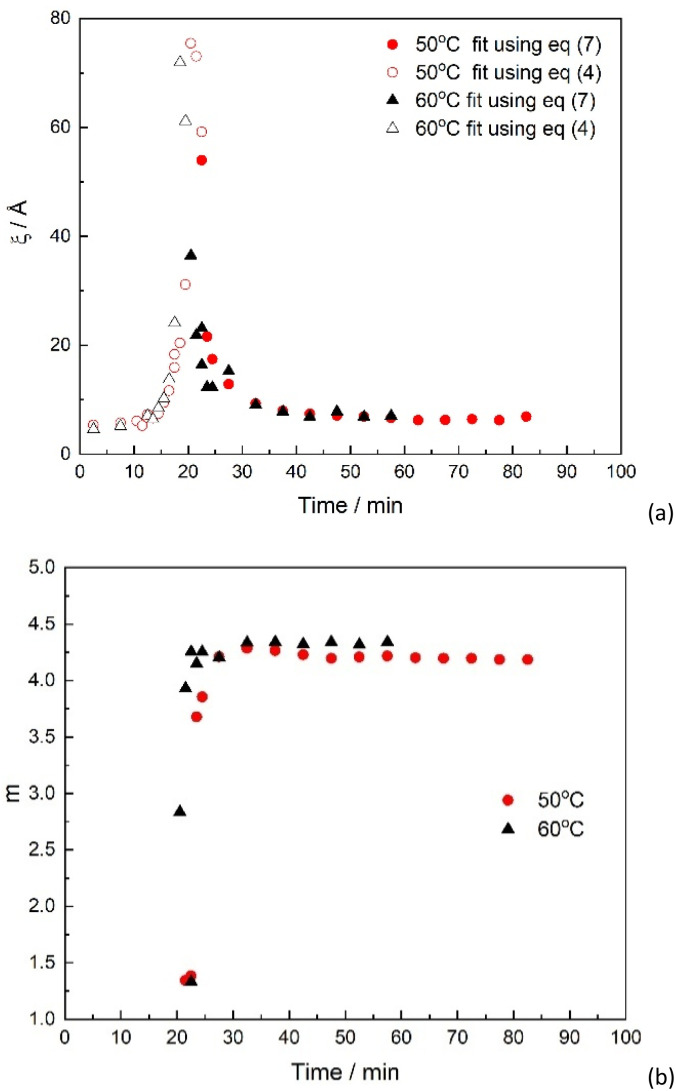
Fitting parameters obtained from the SANS data of EpdIL cured at different temperatures. (a) correlation length, *ξ* and scale parameter *I*_OZ_(0) of the Lorentzian component as obtained from fits using eqn (4) (empty symbols) and eqn (7) (filled symbols); (b) Porod exponent (*m*) obtained from the fits using eqn (7).

Curve fitting using eqn (7) was successful for all scattering curves for both temperatures, 50 °C and 60 °C. The change in the fitting parameters is presented in Fig. 7. As expected, the values of *ξ* rise faster for the reaction carried out at higher temperatures and achieves higher values (Fig. 7(a)).

The maximum value of the Porod exponent, *m*, was not noticeably affected by the temperature of the curing reaction, reaching values well above 4, as discussed in earlier sections (Fig. 3(c) and 7(b)).

### SANS characterisation of the block-copolymers

Addition of block-copolymers to epoxy formulations is known to produce nanostructured materials.^57,58^ In the recent study,^13^ the self-assembly of multifunctional block-copolymers (MF-bcP) was exploited to create a finer biphasic hierarchical microstructure. As shown in Scheme 1, one of the blocks of the MF-bcP has an oxirane ring and is compatible with DGEBA, whilst the other block contains an anion identical to that in the ionic liquid, *i.e.* EMIM-TFSI and compatible with it.

**Fig. 8:**
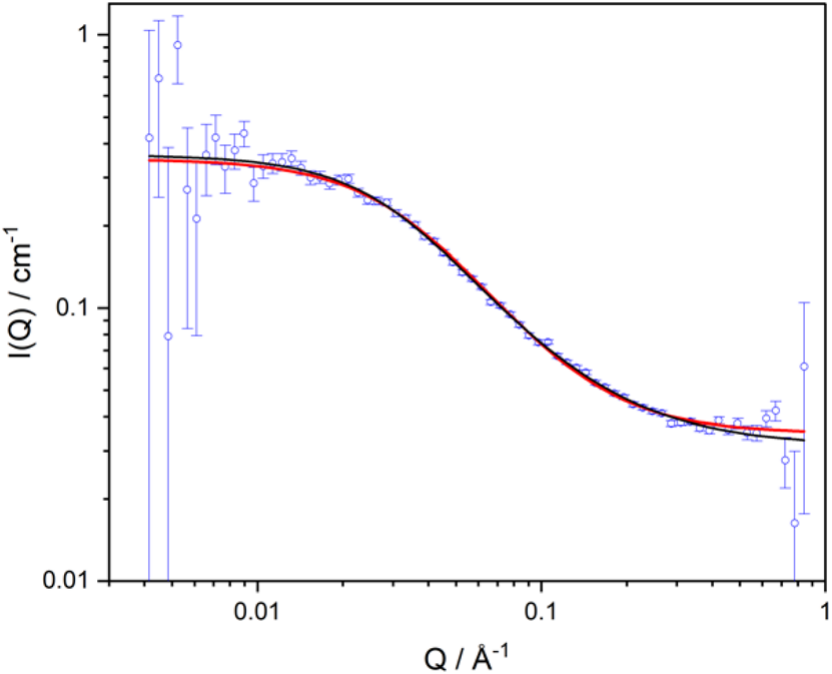
SANS data for MF-bcP in the d-IL. The lines are fits using eqn (8) and a form factor as given by eqn (12) with a fixed value of polydispersity equal to 2 (red line) and eqn (15) (black line).

The scattered intensity of a solution of MF-bcP (10 mg ml^−1^) in deuterated IL is plotted in Fig. 8. The scattering data provide a measure of the conformation of the block copolymer. Earlier, using TEM, it was shown that the MF-bcP forms micelles in IL solutions with a diameter of 23 ± 9 nm using an initial concentrations of 10 mg ml^−1^.^13^ The TEM images were taken after staining sample with iodine over 2 days, the SANS measurements were done over significantly shorter period of time which was clearly insufficient for micelles formation.

For dilute solutions, the structure factor S(*Q*) in eqn (2) tends to unity and can be neglected. The differential scattering cross section dΣ(*Q*)/dΩ (eqn (2)) is therefore given by:8
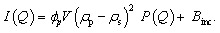


The volume of the polymer coil *V* can be written as:9
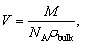
where *M* is the molecular weight of the polymer, *N*_A_ is Avogadro's Number, and *ρ*_bulk_ is the polymer bulk density.

For monodisperse Gaussian chains the variation of the scattered intensity with scattering vector *Q* described by the form factor *P*(*Q*) is modelled by the Debye equation:10
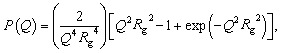
where *R*_g_ is the radius of gyration of the polymer which is related to its degree of polymerization *N* and statistical segment length *a* as follows:11
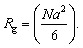


For a polydisperse system in a *θ* solvent, the scattered intensity is described by assuming a Schulz–Zimm type molecular weight distribution and the form factor is given by a modified Debye equation:^59^12
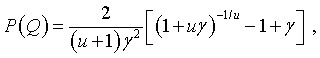
where *u* = *M*_w_/*M*_*n*_ − 1 and *γ* = (*Q R*_g_)^2^/(1 + 2*u*).

Since the scattered intensity at *Q* = 0 is given by:13
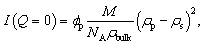
it is possible to check whether the fits correspond to the single chain scattering. Fitting parameters using eqn (12) and (13) are given in Table 2. Using eqn (13), the molecular weight of the block copolymer (M) can be calculated from the extrapolated value of the scattered intensity at *Q* = 0, and the contrast. M was found to be equal to 8739 g mol^−1^. The chemical formula of the p(GMA)_36_-*block*-*co*-p(DMAEMA-TFSI)_7_, with the molecular weight of the GMA block is 5112 g mol^−1^ and for the DMAEMA-TFSI it is 3360 g mol^−1^. Therefore, the calculated *M*_w_ value of the block copolymer is 8472 g mol^−1^, close to the experimental value of 8739 g mol^−1^.

**Table 2:** SANS fitting parameters for MF-bcP using eqn (8) and different expressions for the form factor *P*(*Q*) as indicated

	*P*(*Q*), eqn (10)	*P*(*Q*), eqn (15)
*I*(*Q* = 0)/cm^−1^	0.317 ± 0.0056	0.333 ± 0.0083
*R*_g_/Å	46.3 ± 0.7	46.7 ± 1.4
*M*_w_/*M*_*n*_	2 (fixed)	na
*B*_inc_/cm^−1^	0.0348 ± 0.0003	0.0313 ± 0.0006
Porod exponent, *m*		1.58 ± 0.04

Excluded volume effects are not considered by the simple Debye equation (eqn (10)), thus an additional Flory exponent, *ν*, in an adjusted equation for linear polymers was introduced by Benoit^60^ resulting in:14
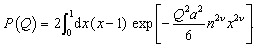
Here the parameter *ν* represents the excluded volume which is related to the Porod exponent *m* (*ν* = 1/*m*). This expression was converted into an analytical form by Hammouda and the model is included in the analysis program SasView.^61^ It makes use of the form factor:15
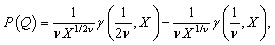
where *X* = *Q*^2^*R*_g_^2^(2*ν* + 1)(2*ν* + 2))/6 and 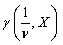 is the incomplete gamma function:16
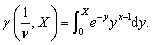
When the polymer chains follow Gaussian statistics (*ν* = 0.5), eqn (15) simplifies to the Debye model (eqn (10)). The result of fits using the polymer with excluded volume form factor are listed in Table 2 (3rd column). The excluded volume exponent is 0.67, suggesting chain expansion in the IL solvent. The fit is shown in Fig. 8.

## Effect of block copolymer on structural changes during RIPS

Previously it was shown^13,62^ that addition of a block copolymer to epoxy based formulations causes a reduction in the size of the characteristic structures. In the epoxy based formulations studied here, addition of MF-bcP led to formation of additional structures in the range of 50 nm, with the longer length scale microstructure associated with the epoxy reverted to the fused nodule structure, resulting in the reduction of the Young's modulus.^13^

**Fig. 9:**
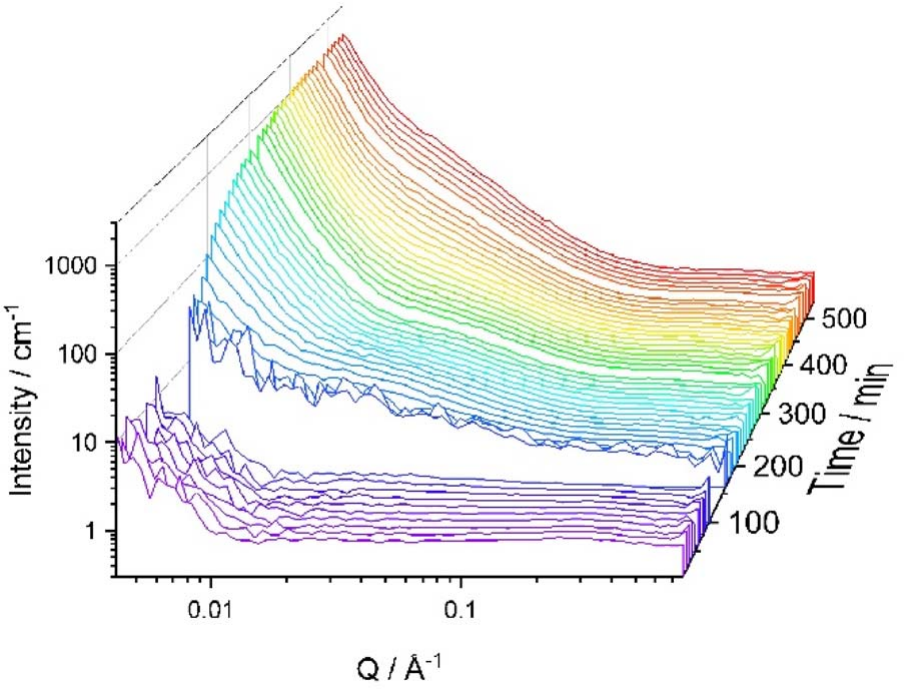
SANS data for Ep/MF-bcP, during curing in a mixture d- IL:h-IL (50 : 50 wt%).

The SANS data of curing formulations with the addition of 1 wt% MF-bcP is reported in Fig. 9. The experiment was carried out using SANS2D in the minimum Q-range of 0.0015 Å^−1^, corresponding to *d*-spacings of 4187 Å^−1^. Similar to the epoxy resin formulations without block-copolymer (Fig. 1), the SANS data display two structural phases of different length scale. No changes in the SANS curves were observed in the first two hours of curing, at room temperature. After *ca.* 180 minutes, a change in the scattering pattern at *Q* > 0.01 Å^−1^ between samples with and without block-copolymer became evident, especially in the mid-range of *Q* values (0.01 < *Q* < 0.1 Å^−1^).

It can be seen from Fig. 1 and 6 that for all samples without block-copolymer and independent of the curing temperature, the shoulder in the midrange of *Q* values (0.01 < *Q* < 0.1 Å^−1^) is clearly visible identifying the start of the formation of heterogeneities. As reaction progresses, for samples without MF-bcP, this shoulder begins to disappear and a sharp upturn of the scattered intensity occurs at *Q* < 0.05 Å^−1^, as discussed earlier. However, upon addition of the 1 wt% MF-bcP, the position and intensity of the midrange shoulder remain largely unchanged with the reaction time. This indicates that initially formed structures are present in the fully cured samples which is in good agreement with the SEM images.^13^

The main noticeable difference between data in Fig. 1 and 9 is in the intermediate *Q* region from 0.01 to 0.10 Å-1. The scattering shoulder within this *Q* range mainly grows in intensity with curing time indicating that the domain size, after an initial increase, remains nearly constant.

**Fig. 10:**
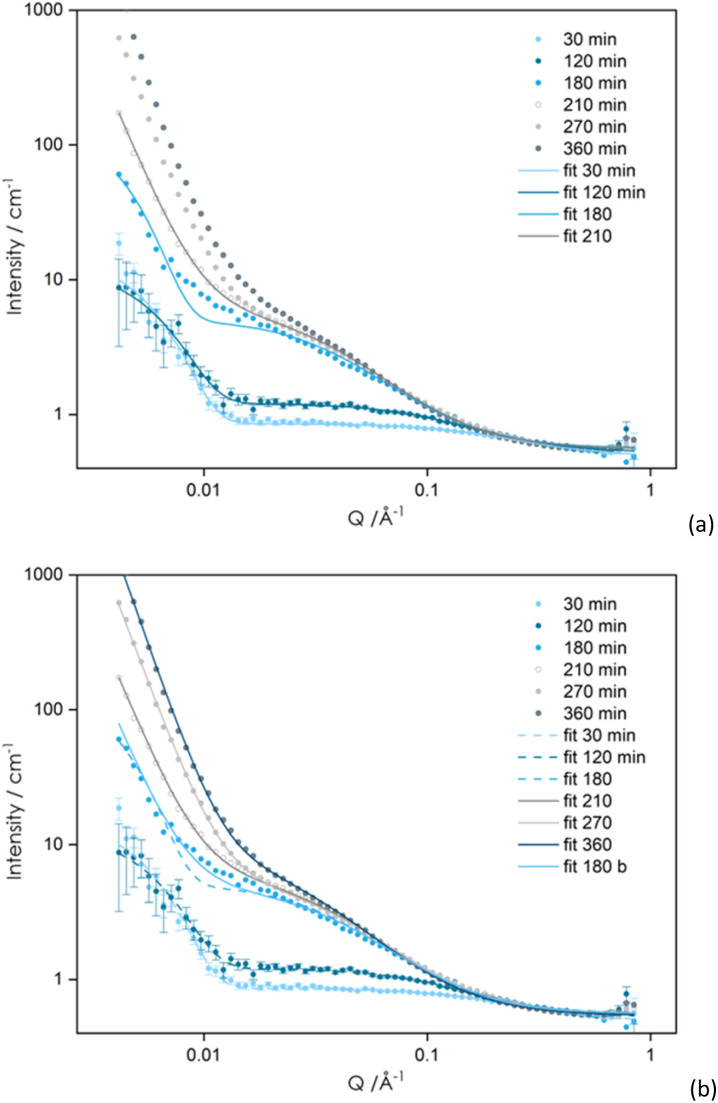
SANS data for Ep/MF-bcP at different curing times, in a presence of a mixture of d- IL:h-IL (50 : 50 wt%). The lines are fits to the experimental data using (a) eqn (4) and (b) eqn (7).

The SANS data presented in Fig. 9 were fitted to eqn (4) and (7), as for formulations without MF-bcP, and presented in Fig. 10.

**Fig. 11:**
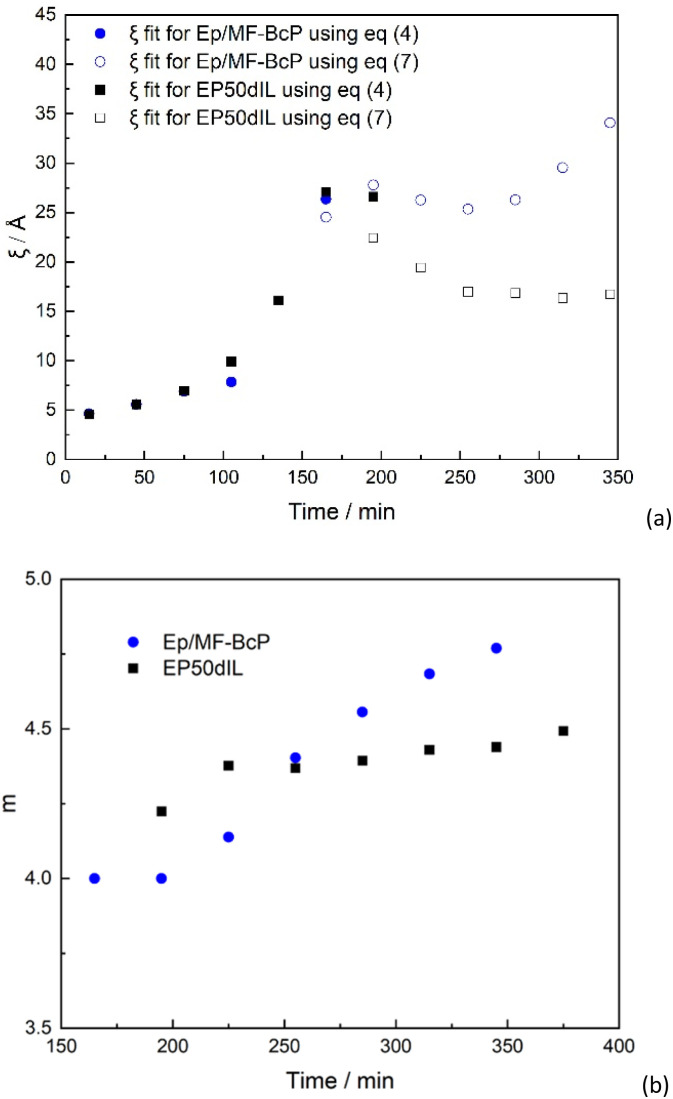
Time dependence (a) of the correlation length of the liquid-like fluctuations as obtained from the SANS data for formulations with and without MF-bcP at different curing times and (b) Porod exponent.

Comparison of the time dependence of the *ξ* correlation length for formulations with and without MF-bcP is given in Fig. 11(a). The presence of the block-copolymer alters the rate of change of the correlation lengths. In the first stages of the curing process, the time dependence of *ξ* is the same as the one observed in the absence of the block-copolymer (Fig. 11(a)). For *t* > 180 min, however, rather than a decrease, *ξ* for system containing MF-bcP remains approximately constant, with an average value of 25 Å up to at least 300 min.

## Conclusions

In this work, the curing kinetics of epoxy-based formulations were investigated. By curing the epoxy in the presence of deuterated ionic liquid, using SANS, it was possible to monitor the evolution of different structural features: (a) the initial cluster formation at short times and (b) the highly cross-linked 3D network structure which develops upon further curing.

All formulations initially consisted of a single phase but, as curing progressed, small clusters were formed. The evolution of these clusters was monitored as a function of time. The size of the clusters, represented by the short correlation length, *ξ*, was observed to increase with maximum values ranging from 25 to 75 nm, depending on the temperature. The number of clusters also increased at first followed by a decrease in parallel to formation of larger and more complex structures. Increasing the curing temperature resulted in the increase of the reaction and phase separation rates leading to formation of larger structures at short times. Addition of the multifunctional block copolymer to the solution of the epoxy in ionic liquid led to formation of a hierarchical structure, earlier observed *via* SEM. Although the SANS data showed structural differences compared to the epoxy samples without block copolymer, this did not affect the rate of the curing.

## Author contributions

NS: conceptualization; investigation; formal analysis; funding acquisition; methodology; writing and editing. LPC: investigation; formal analysis; funding acquisition; methodology; editing. SY: Resources; editing. VA: conceptualization; investigation; formal analysis; funding acquisition; methodology; writing and editing. All authors have given approval to the final version of the manuscript.

## Conflicts of interest

There are no conflicts to declare.

## Supplementary Material

RA-015-D5RA05789B-s001

## Data Availability

SANS data for all formulations discussed in this manuscript is available at https://doi.org/10.5286/ISIS.E.RB2000031; https://doi.org/10.5286/ISIS.E.RB2220051; https://doi.org/10.5286/ISIS.E.RB2320239. Supplementary information (SI) is available. See DOI: https://doi.org/10.1039/d5ra05789b.
