# Supplementary material for: Study of microstructure formation in epoxy based systems using small angle neutron scattering
Source: RSC Adv. 2025 Dec 17;15(59):50844–55. doi: 10.1039/d5ra05789b (PMC12710605; doi:10.1039/d5ra05789b)
Supplement: RA-015-D5RA05789B-s001 [file RA-015-D5RA05789B-s001.pdf]

Supplementary Information (SI) for RSC Advances.
This journal is © The Royal Society of Chemistry 2025

Supplementary Information

Microstructure formation in epoxy-based systems studied using Small Angle Neutron Scattering

Natasha Shirshova,*[a] Leide Cavalcanti,[b] Sarah Youngs,[b] and Valeria Arrighi[c]

[a] Department of Engineering, Durham University, South Road, Durham, DH1 3LE, United Kingdom
[b] Science Technology Facilities Council; Isis Neutron and Muon Source; Harwell Campus; Oxfordshire; OX11 0QX, United Kingdom
[c] Institute of Chemical Sciences, School of Engineering & Physical Sciences, Heriot-Watt University. Edinburgh, EH14 4AS, United Kingdom

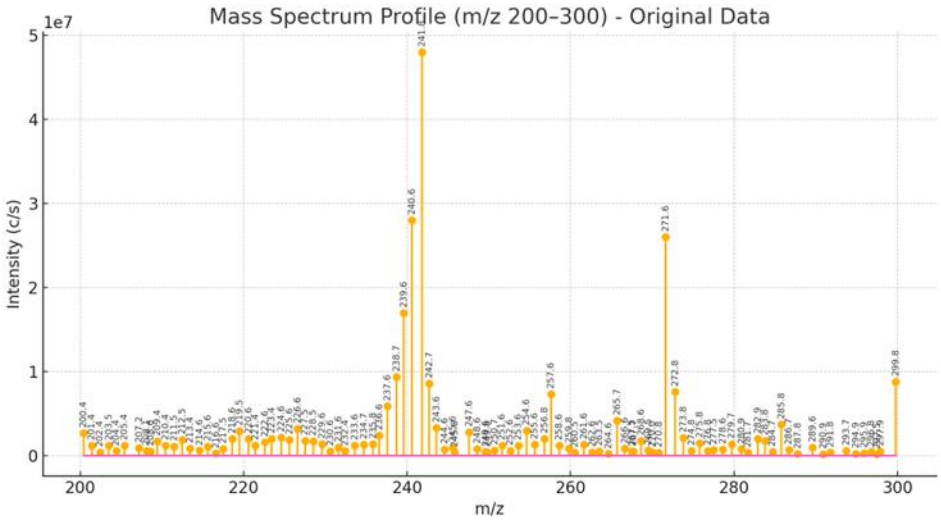

Figure S1. Mass Spectrum Profile (m/z 200–300) of the deuterated IL with Peak Labels.

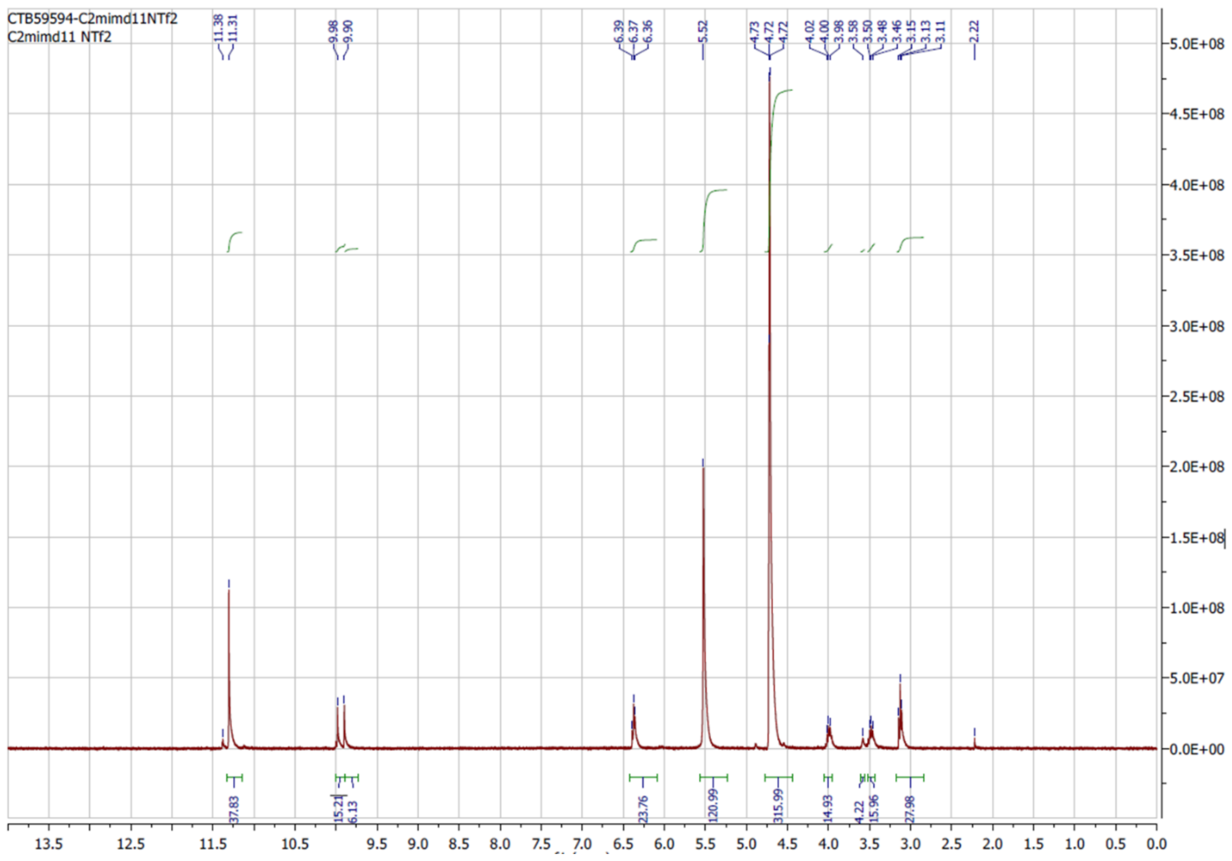

Fig S2. 1H NMR spectra of the d-IL in d-DMSO.

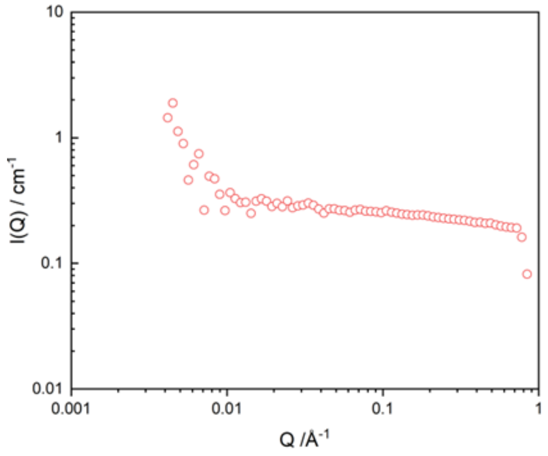

Figure S3. SANS curves for a block copolymer solution in h-IL.

Table S1. SDL values for compounds used

Chemicals	Formula	SDL/ $Å^{-2} \times 10^6$
DGEBA	$C_{21}H_{24}O_4$	1.29
iPDA	$C_{10}H_{22}N_4$	0.657
IL	$C_8H_{11}O_4N_3F_6S_2$	2.38
d-IL	$C_8D_{11}O_4N_3F_6S_2$	4.88

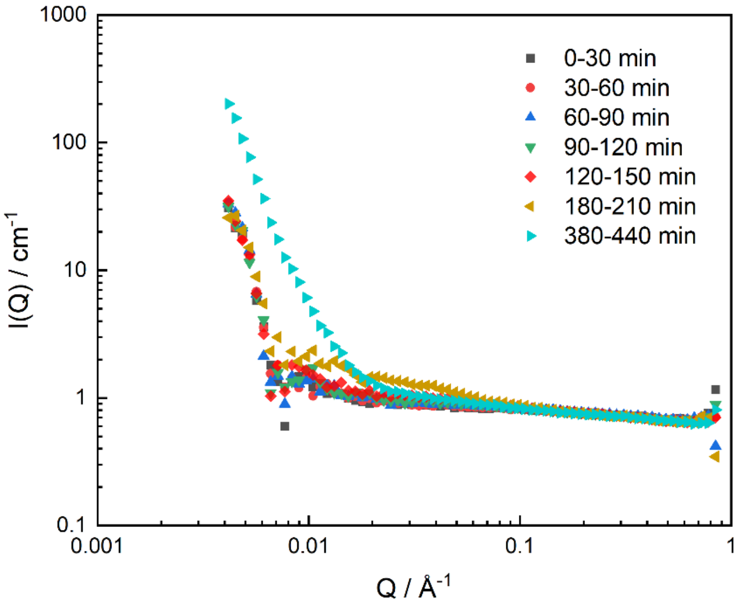

Figure S4. SANS data for EPhIL as a function of curing time. The sample consists of epoxy, iPDA in a h-IL. EP:iPDA = 4:1 wt./wt.; [IL]= 40 vol%.
